# THE COST OF BEING A DOCTOR

**DOI:** 10.1590/0102-672020180001e1368

**Published:** 2018-07-02

**Authors:** Bruno ZILBERSTEIN, Osvaldo MALAFAIA, Nicolau Gregori CZECZKO

**Affiliations:** 1 ex-Presidents Brazilian College of Digestive Surgery - CBCD; 2 President Brazilian College of Digestive Surgery - CBCD

The qualities that make the doctor and the surgeon a successful professional is intense curiosity, warmth capacity and professional training with the art of making proper diagnosis and surgical intervention.

Most of us go to medicine or embrace it with a missionary spirit, committed to helping people.

We are grateful for the opportunity to help, proud of our ability to make a good diagnosis, designate and perform the appropriate treatment, and have a great reward for the trust we have received from our patients. We are imbued and exercising a profession not a business.

According to the American College of Surgeons - ACS - a profession or a professional is considered to have: 1) the domain of the use of a specific knowledge; 2) relative autonomy of professional practice and the privilege of self-examination; 3) to act altruistically serving individuals or society; 4) have the responsibility to maintain and expand their knowledge and skills; 5) on the other hand, they are charged and have the duty to put the needs of patients above theirs and to have always exemplary behavior.

If these concepts and this model were virtuous in the last century, how should be our surgeon in the 21^st^ century?

The progress of the past 50 years has brought tremendous advancements to medicine in general and to surgery in particular. While on the one hand this was beneficial to the whole, on the other hand the surgeon in particular had to specialize more and more and his knowledge needed to be increased and restricted to specific areas of knowledge.

That is how he became more and more distant from the general surgeon, restricted to individualized areas such as cardiac, vascular, head and neck surgeries. That´s why the surgery of the digestive tract emerged 30 years ago, and now it also migrates to subspecialties, such as surgeries of upper digestive tract, hepatobiliopancreatic, transplants - liver, pancreas and intestine - and more, bariatric surgery, videosurgery, robotics and oncological surgery, in addition to the traditional coloproctological surgery.

These areas and subspecialties often cause conflict between professionals and competition, not often salutary. And in this context it is added that professional autonomy is increasingly difficult to be exercised, since the surgeon is increasingly becoming a professional contracted by health operators or hospital institutions^3^.

It is no longer new hearing from the patients that they were attended or operated in “such” hospital, not even knowing the name of the professional or medical team that attended them. This makes or leads the surgeon to abide rules, regulations or protocols that do not always match the best treatment or best practice that his knowledge and experience allows him to offer^1^. This also made the surgeon more and more obliged to perform bureaucratic activities and undergoing in a series of tasks that have nothing to do with the execution of the medical act itself. According to Woolhandler et al.^5^, the surgeon spends at least 20% of his time in bureaucratic activities that add nothing to professional performance.

The doctor begins to be evaluated by little scientific concepts, such as the satisfaction of the patient and the opinion and the scrutiny of the administrative and lay body of the hospitals; is no longer evaluated by his success, nor by the references in successful treatments and by academic or professional performance^4^. This all discourages and makes lose interest even in the professional practice. We are being qualified and evaluated by questionnaires of patients ‘and institutions’ satisfaction for their own interests^1^. And this is one more reason why surgical specialties are becoming less and less sought by young people in training.

And what to say in our country with the creation of a exaggerated number of medical schools forming “who knows what”, taught “who knows by whom”!

All this led to the syndrome known as “burnout”, characterized by physical and emotional exhaustion after a long period of work. Characteristics include emotional exhaustion, cynicism, disinterest, and frustration with personal fulfillment^2^.

Allied to all this, it is worth mentioning that the doctor often receives or is paid with values ​​lower than the cost of a haircut! This fact was even emphasized by a senior executive leader of a major operator, who characterized the doctor as a being “differentiated”, “cheap” and “easy to find anywhere”!

And how to improve this deplorable situation? With human relations, teamwork, case discussion, shared responsibility and associative life. It is at this moment that class associations come into play.

Specialty societies have to give and offer the necessary backing for good professional performance. Allow and assist the surgeon to exercise his profession with dignity and safety. Support and defend him in professional performance. Offering opinions that support decent work, reinforcing and highlighting professional proficiency.

In this sense, we would like to emphasize the mission of the Brazilian College of Digestive Surgery, which in its 30 years of life has always been concerned with the dignity of medical practice. We want to point out that was thanks to the CBCD that our specialty was recognized. The CBCD’s actions with the AMB enabled the CBHPM to be implemented and its values ​​permanently updated. CBCD certified the actuation areas in digestive endoscopy, videosurgery and bariatric surgery. It provided a medical journal to maintain surgeons actualized - today being one of the most important in Brazilian surgery. It organized and made available a series of “lato sensu” graduate courses that allow the constant updating of his associates. It offers the possibility of exchange of opinions and discussions of cases, as well as legal advice when necessary. It is CBCD’s concern the well-being of our surgeons and, for this, he has been fighting incessantly dignifying and giving more and more value to the medical practice.

As final message of this critical analysis of the 21^st^ century digestive tract surgeon, we would say that each one of us has to fight to dignify and to make recognized our performance in the medical hospital environment, using more and more the class associations to support our actions in favor of the ethical and categorical exercise of our professional activity.
